# Supplementary material for: Multiomic Profiling Identified EGF Receptor Signaling as a Potential Inhibitor of Type I Interferon Response in Models of Oncolytic Therapy by Vesicular Stomatitis Virus
Source: Int J Mol Sci. 2022 May 8;23(9):5244. doi: 10.3390/ijms23095244 (PMC9102229; doi:10.3390/ijms23095244)
Supplement: Supplementary file 1 [file ijms-23-05244-s001.zip › Figures S1-S3.pdf]

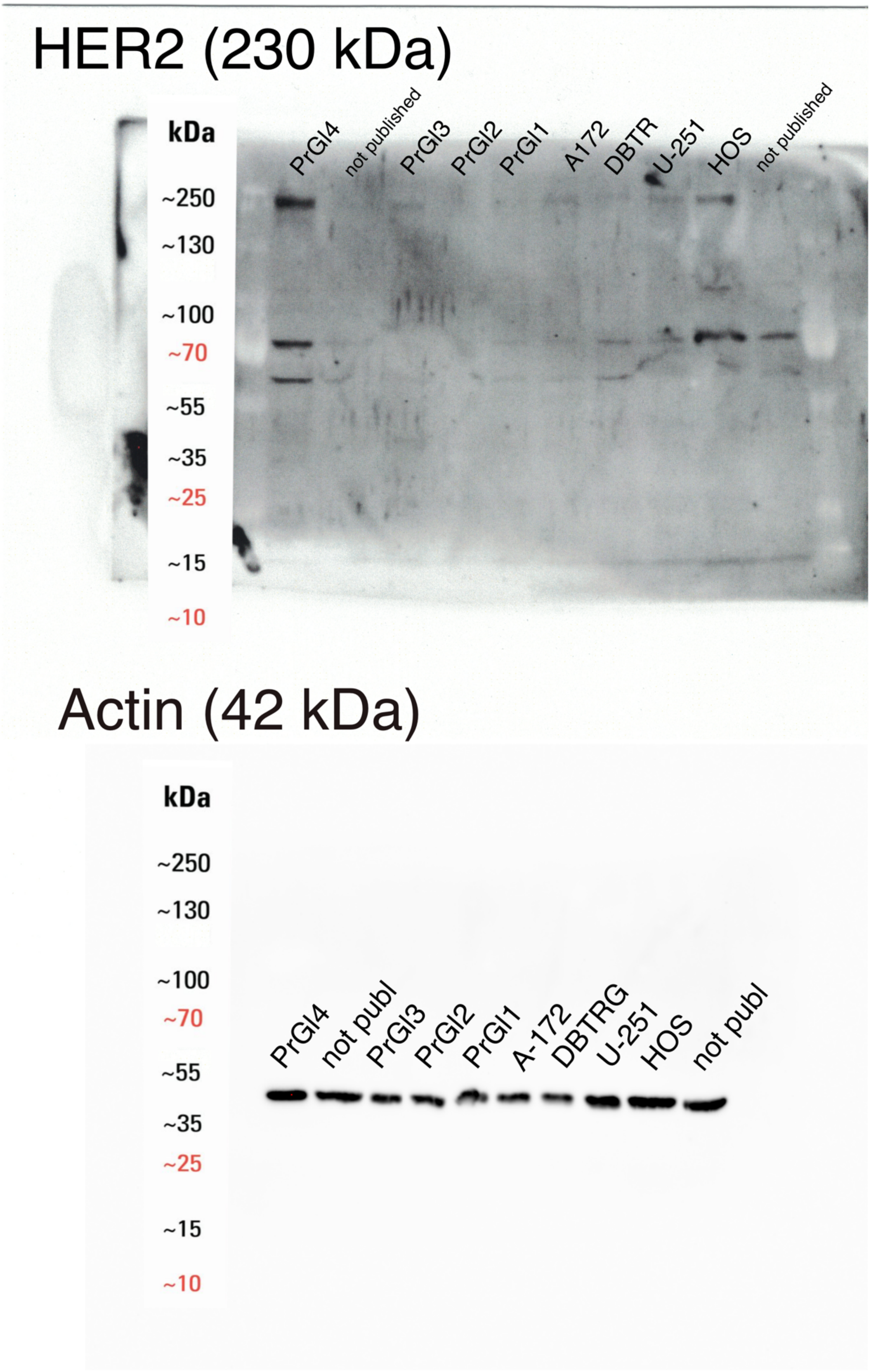

Figure S1. Western blot analysis of HOS, U-251 MG, and DBTRG-05MG cells. Uncropped western blot scan.

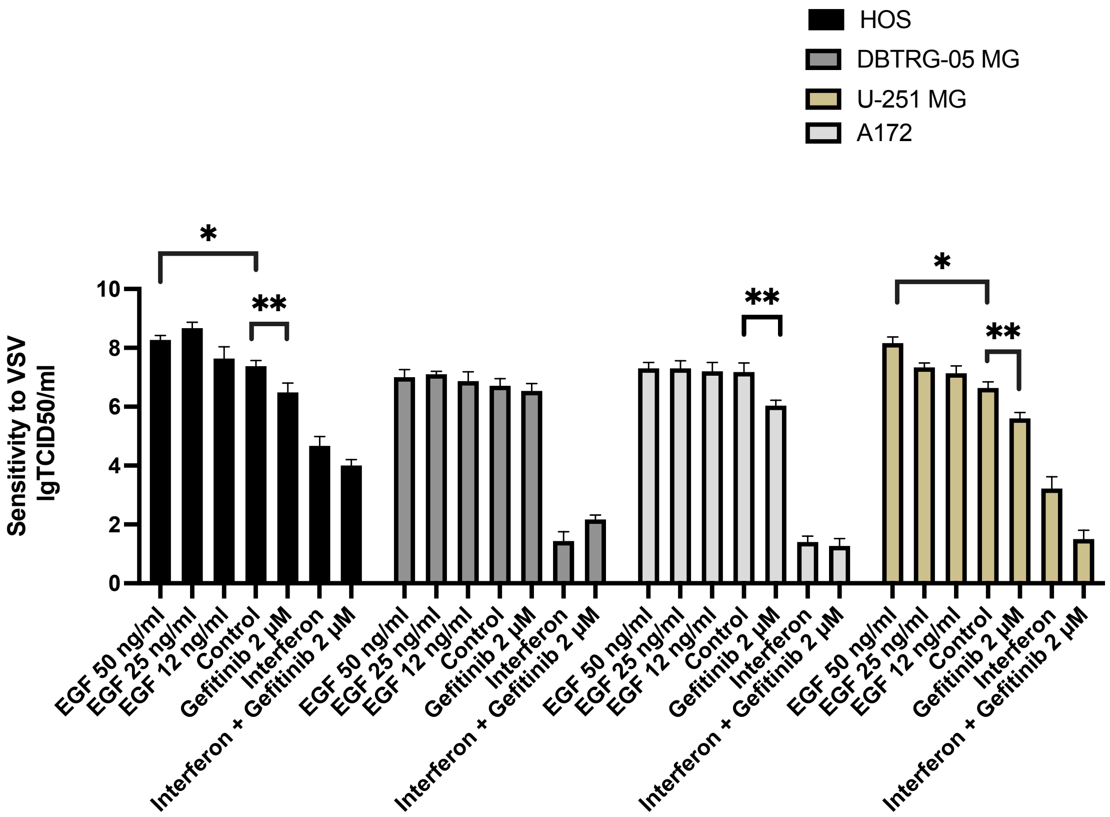

Figure S2. Sensitivity of model cell lines to VSV after treatment with gefitinib (2 μM), interferon α2β (1000 U/ml), EGF (50, 25 or 12 ng/ml) and simultaneous treatment of gefitinib and interferon for 24 h. Gefitinib treatment has shown synergic effect with interferon treatment in inhibition of viral production in U-251 MG and HOS cells (n = 3 replicates per group). Bars represent mean values with SD, * $p < 0.05$, ** $p < 0.01$, t-test).

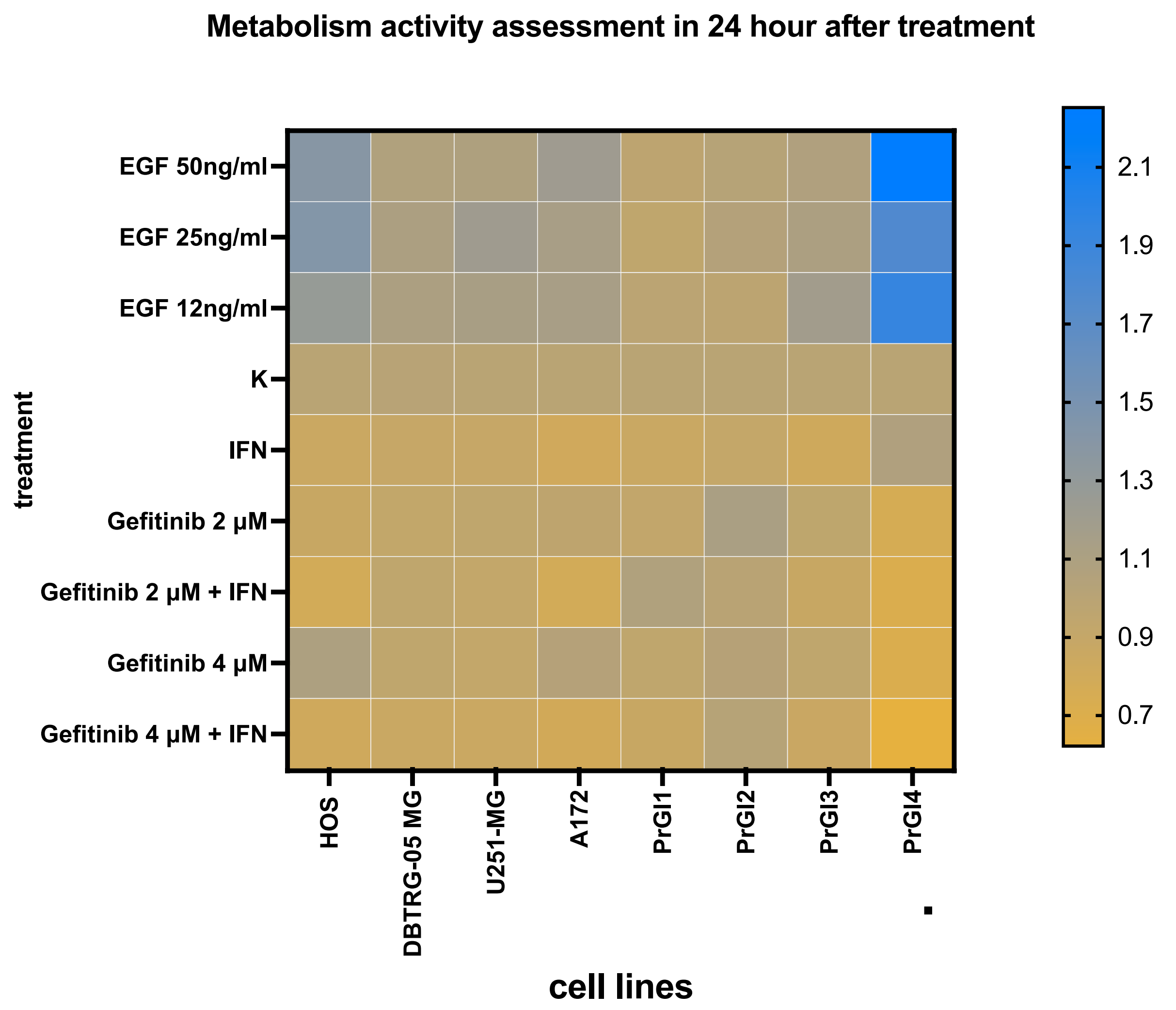

Figure S3. Metabolic activity assessed by MTT assay dependence on EGF, Gefitinib concentration or interferon presence after 24 hours of incubation. The experiment was performed in sextuplicate from three independent experiments. Absorbance was determined as the average of data for each experiment, nontreated cells were used as controls, considering 100% metabolic activity.
