# Supplementary material for: Multiomic Profiling Identified EGF Receptor Signaling as a Potential Inhibitor of Type I Interferon Response in Models of Oncolytic Therapy by Vesicular Stomatitis Virus
Source: Int J Mol Sci. 2022 May 8;23(9):5244. doi: 10.3390/ijms23095244 (PMC9102229; doi:10.3390/ijms23095244)
Supplement: Supplementary file 1 [file ijms-23-05244-s001.zip › File_S1.pdf]

Supplementary File S1. Attempting transfection of DBTRG-05MG cell line to provide overexpression of HER2 gene

1. Transfection by the lentiviral vector.

Cloning the HER2 gene to the lentiviral vector (pL-CMV-HER2-puro) was performed as follows. For the production of lentiviral particles, plasmid constructs pREV, pGAG-pol, p-VSV-G, and pL-CMV-HER2-puro have been used that contain HIV-1 package proteins. In addition, the pL-CMV-eGFP-puro vector was used as a control in parallel experiments. HEK293T cells were plated on Petri dishes up to 35% confluence. Afterward, they were transfected by packaging plasmids (on 2:1:1:4 ratio, respectively). After 16 h, culture media was refreshed to DMEM containing 2% FBS. After 8 h, a first stock solution was collected. After that, lentiviral particles were collected twice a day. Viral particle titer was estimated on 5 pooled stock solutions containing the control vector, using a method of final dilutions. The lentivirus titer was not lower than 10^7, which made an effective production of HER2 vector particles feasible. However, after transduction of DBTRG-05MG cells of interest, a puromycin selection (1.5 μg/mL) demonstrated a very low percentage of transduced cells. Furthermore, survived cells showed no increase in HER2 mRNA expression. Presumably, the failed transduction was caused by the large size of the integration cassette (ca. 5000 bp), which was a possible upper boundary for lentiviral vectors.

2. Transient transfection by pcDNA3 vector.

Overexpression of HER2 in the DBTRG-05MG cell line was attempted to reach by transient transfection. The plasmid expressing wild-type human HER2 (Addgene, USA, cat.# 16257) was used [52].

Five methods of transfection were used.

Calcium phosphate transfection was performed according to Kwon and Firestein [53]. For treatment of 6 mln cells, 20 μg DNA was used.

Turbofect (Thermo Fisher, USA) transfection reagent was generally used according to the manufacturer's protocol. For treatment of 6 mln cells, 24 μg DNA and 50 μL Turbofect were used.

Lipofectamine 3000 (Thermo Fisher, USA) transfection reagent was also used generally according to the manufacturer's protocol. To treat 6 mln cells, 12 μg DNA, 30 μL Plus Reagent, and 25 μL Lipofectamine were used.

Polyethylenimine (PEI) transfection was done according to Fukumoto et al. [54]. To treat 6 mln cells, 20 μg DNA and 100 μg PEI were used.

Finally, electroporation was used for transfection. Cells (2 mln) were treated in 2 mm cuvette at 200 V and 600 mF. Optimal conditions were selected by variation of DNA quantity from 20 to 30 μg. PBS or DMEM were used as a variation of media. The best result of eight used protocols was reached at 200 V, 500 mF, 30 μg DNA, DMEM medium, squared pulse.

To control optimal transfection, red fluorescent protein (RFP) was used as a transgene under CMV promoter. FACS was used to estimate of transfection percentage. Briefly, fluorescent cells were quantified using BD LSR Fortessa cytofluorimeter (Beckman Dickinson, USA). Data were processed using FACS Diva software. RFP emission was detected in the P.E. channel. Experiments were done in three biological replicates, each, in turn, in three technical replicates.

Results for five methods are represented in Table S5. As one can see from this Table, the DBTRG-05MG cell line was shown to be very resistant to the transfection of any method compared to the HEK-293T control. The HER2 plasmid would transfect DBTRG-05MG even worse, as the transgene consists of 3700 bp (cf. 700 bf for RFP).

In conclusion, two approaches for transfection, lentiviral and plasmid vectors, failed to provide overexpression of HER2 in DBTRG-05MG glioma cell lines. In the model used in our study, this task was not technically feasible.

Table S5. Transfection efficiency of DBTRG-05MG cell lines by the red fluorescent protein (RFP) plasmid

**Method**	**Percentage of RFP positive cells**	
	**DBTRG-05MG cell line**	**HEK-293T cell line**
Calcium phosphate transfection	4.6%	89%
Turbofect	1.3%	47%
Lipofectamine 3000	6%	92%
PEI	4.6%	72%
Electroporation	2.7%	68%
